# The Magnitude of Anesthesiology Residents Burnout at Shahid Beheshti University of Medical Sciences: A Cross-Sectional Study

**DOI:** 10.5812/aapm-159987

**Published:** 2025-03-08

**Authors:** Maedeh Karimian, Ali Dabbagh, Parisa Sezari, Kamal Fani, Marzieh Shahrabi, Alireza Shakeri

**Affiliations:** 1 Anesthesiology Research Center, Shahid Beheshti University of Medical Sciences, Tehran, Iran; 2 Department of Anesthesiology, Anesthesiology Research Center, Shahid Modarres Hospital, School of Medicine, Shahid Beheshti University of Medical Sciences, Tehran, Iran; 3 Department of Anesthesiology, School of Medicine, Shahid Beheshti University of Medical Sciences, Tehran, Iran

**Keywords:** Anesthesiology Residency Program, Depressive Disorder, Professional Burnout, Quality of Life

## Abstract

**Background:**

Burnout is a psychological syndrome characterized by emotional exhaustion, depersonalization, and reduced personal accomplishment, particularly prevalent among anesthesiology residents due to their demanding work environment.

**Objectives:**

This study aimed to assess the prevalence of burnout and its associations with depression, sleep quality, and quality of life among anesthesiology residents at Shahid Beheshti University of Medical Sciences.

**Methods:**

A cross-sectional study was conducted in 2023 with 51 anesthesiology residents. Validated Persian versions of standardized tools, including the Maslach Burnout Inventory (MBI), Patient Health Questionnaire-9 (PHQ-9), Epworth Sleepiness Scale (ESS), STOP-Bang test, and World Health Organization (WHO) Quality of Life (WHOQOL-BREF) Questionnaire, were used to measure burnout, depression, sleep quality, and quality of life. Descriptive statistics and correlation analyses explored associations between these variables.

**Results:**

Burnout was highly prevalent: 41.2% of residents experienced severe emotional exhaustion, 66.7% exhibited high depersonalization, and 100% reported reduced personal accomplishment. Severe depression affected 17.65% of participants, correlating strongly with all burnout dimensions. Emotional exhaustion was significantly associated with increased daytime sleepiness (r = 0.470, P < 0.001), while burnout severity inversely impacted physical, psychological, and social quality of life. Emotional support emerged as a critical protective factor against burnout. Exploratory analyses revealed no significant gender differences in burnout, depression, or sleep quality, though small effect sizes suggested trends toward higher emotional exhaustion and depression among female residents.

**Conclusions:**

The study highlights the alarming prevalence of burnout among anesthesiology residents, driven by occupational stress, sleep disturbances, and mental health challenges. These findings align with research in other high-stress specialties, such as emergency medicine and surgery, but also underscore unique stressors faced by anesthesiology residents. Targeted interventions, such as optimizing work conditions, enhancing emotional support, and addressing mental health and sleep issues, are urgently needed. Longitudinal and comparative studies are recommended to further explore burnout progression and develop specialty-specific strategies to improve resident well-being and patient care outcomes.

## 1. Background

Burnout, first described by Herbert in 1974, is a psychological syndrome resulting from chronic workplace stress, characterized by emotional exhaustion, depersonalization, and reduced personal accomplishment (1). The World Health Organization (WHO) further defines burnout as a "state of vital exhaustion" marked by feelings of ineffectiveness, particularly in caregiving professions (2). Physical symptoms, such as headaches, fatigue, and sleep disturbances, often accompany psychological manifestations like irritability, decreased concentration, and even substance abuse (3). These symptoms can severely impair work effectiveness and personal well-being, making burnout a critical concern in high-stress fields such as healthcare.

Anesthesiology residents, in particular, face heightened risks due to the demanding nature of their work, including long hours, frequent night shifts, and the life-critical responsibilities inherent to their specialty (4, 5). In Iran, burnout among anesthesiology residents is exacerbated by unique systemic, societal, and cultural challenges. Long working hours, mandatory post-graduation service obligations, and societal expectations contribute to high levels of occupational stress (6, 7). The healthcare system is further strained by a significant emigration of physicians — over 4,000 doctors left Iran in 2022 — and a shortage of residency positions, with 200 to 300 vacancies reported (6). Financial pressures also play a role, as residents must pay a fee of approximately $6,000 to bypass compulsory service, adding to their burden (6, 8). These factors have led to alarming outcomes, including 16 reported suicides among resident doctors within a 9-month period, underscoring the urgent need to address burnout in this population (6, 9). Additionally, societal pressures and cultural expectations often leave residents feeling unsupported, further intensifying their emotional and psychological strain (7).

The present study uniquely contributes to the literature by examining burnout among Iranian anesthesiology residents, a population grappling with structural challenges such as mandatory service obligations, physician emigration, and cultural stressors (6, 7).

## 2. Objectives

By investigating the interplay between occupational demands, mental health (e.g., depression), sleep quality, and quality of life, the present study aims to identify context-specific drivers of burnout and propose tailored interventions. Such insights are critical for addressing the alarming rates of suicide and attrition in Iran’s healthcare workforce while informing policies to improve resident well-being and patient care outcomes.

## 3. Methods

### 3.1. Participants and Data Collection

The study followed the Declaration of Helsinki and was approved by Shahid Beheshti University of Medical Sciences (IR.SBMU.RETECH.REC.1402.363). This cross-sectional study aimed to comprehensively examine the prevalence and determinants of career burnout among anesthesiology residents at Shahid Beheshti University of Medical Sciences in 2023. A total of 51 residents, enrolled in the university’s four-year residency program at affiliated educational hospitals, participated in the study. All participants met the inclusion criteria and provided informed consent, with no exclusions made, as lack of consent was the only exclusion criterion.

Data were collected using a structured online questionnaire that assessed multiple risk factors for career burnout. The questionnaire integrated established scales such as the Persian-translated STOP-Bang test, the Persian-translated Epworth Sleepiness Scale (ESS) Questionnaire, an abbreviated version of the Persian-translated WHO Quality of Life (WHOQOL-BREF) Questionnaire, the Persian-translated Patient Health Questionnaire (PHQ-9) Depression test, and the Persian-translated Maslach Burnout Inventory (MBI), ensuring a comprehensive assessment of participants’ psychological and emotional well-being.

The strict inclusion criteria included all anesthesiology residents, while exclusion criteria were limited to those who expressed dissatisfaction with study participation, ensuring the integrity and representativeness of the sample. Upon obtaining ethical approval, participants were invited to voluntarily complete the questionnaires, with confidentiality and anonymity strictly upheld to safeguard participants' privacy and data integrity.

While self-reported questionnaires may introduce response biases, this study minimized such effects by ensuring participant anonymity, using validated and culturally adapted instruments, and emphasizing the importance of honest and accurate responses during the consent process. The study adhered to ethical principles, with participation requiring informed consent and respect for participants' autonomy. Data collection followed ethical standards, and all data were securely recorded.

This analytical approach aims to determine the prevalence of career burnout and its associated factors, providing valuable insights into the multifaceted nature of career burnout among medical anesthesiology residents. The study’s rationale underscores the pressing need to address career burnout as a pervasive and impactful phenomenon within the medical profession. By employing validated scales and advanced analytical methodologies, this research seeks to elucidate the underlying determinants of career burnout and proposes effective preventive strategies and structural reforms to mitigate its adverse effects and promote the overall well-being of this group of medical professionals.

### 3.2. Measurements

#### 3.2.1. Sleep-Related Indicators

We used the Persian-translated and validated version of the ESS Questionnaire to assess daytime sleepiness (10). The ESS is widely used in the field of sleep medicine as a subjective measure of a healthcare provider's sleepiness. This test contains a list of eight situations in which a tendency to become sleepy on a scale of 0 indicates no chance of dozing, and a tendency to become sleepy on a scale of 3 indicates a high chance of dozing. In general, a total score of 10 or higher is a cause for concern. A score of 16 on the ESS is typically only observed in people with narcolepsy, idiopathic hypersomnia, or moderate to severe obstructive sleep apnea (OSA) (11).

We also used the Persian-translated and validated version of the STOP-Bang test for evaluating OSA, as it may be concomitant with sleepiness (12). This test is widely known as a sensitive, simple, and easy-to-remember screening tool for OSA (13). The STOP-Bang acronym stands for snoring history, tired during the day, observed cessation of breathing while sleeping, high blood pressure, BMI > 35 kg/m^2^ (or 30 kg/m^2^), age > 50 years, neck circumference > 40 cm, and male sex. A score of 0 - 3 indicates a low risk of OSA, a score of 4 - 5 indicates an intermediate risk of OSA, and a score of 6 - 8 indicates a high risk of OSA.

#### 3.2.2. Burnout Indicators

We used the Persian-translated and validated version of the MBI to assess burnout (14). The MBI aligns with the WHO’s 2019 definition of burnout as a legitimate occupational experience that organizations need to address, characterized by three dimensions: Emotional exhaustion, depersonalization, and reduced personal accomplishment (15, 16). This psychological evaluation tool consists of 22 symptom items related to occupational burnout. Across three dimensions, the mean scores offer significant insights into participants’ psychological states. All items in the MBI are evaluated through a 7-point scale based on the frequency of occurrence, ranging from "never" to "daily".

#### 3.2.3. Depression Indicators

The depression test was performed using the Persian-translated and validated version of the PHQ-9 (17). The test is a self-administered validated diagnostic tool with scores ranging from 0 - 27, consisting of nine items, and serves as a scale for depressive symptoms and a diagnostic instrument first introduced in 2001. Its primary function is to evaluate the existence and intensity of depressive symptoms, as well as to identify the potential presence of a depressive disorder. A summation of the individual responses provides a general indication of the severity of depression (18, 19).

#### 3.2.4. Quality of Life Indicators

We used the Persian-translated and validated version of the WHOQOL-BREF Questionnaire (20). These questions examine how a person assesses their overall quality of life, health, and well-being. The assessment comprises 26 items categorized into four primary domains: Physical, psychological, social, and environmental factors. Participants evaluated the items using a five-point Likert Scale, and subsequently, the unprocessed domain scores were converted to a scale ranging from 0 to 100. This transformation reflected higher scores, which are indicative of an elevated quality of life (21, 22).

### 3.3. Statistical Methods

The statistical analysis for this study was conducted using the Statistical Package for the Social Sciences (SPSS) software, version 26.0. Descriptive statistics, including means, standard deviations, medians, and ranges, were calculated to summarize the data obtained from the administered questionnaires. Frequencies and percentages were computed to characterize categorical variables such as gender, marital status, and educational level. Pearson’s correlation analysis was applied to assess relationships between burnout dimensions (emotional exhaustion, depersonalization, personal accomplishment) and other study variables, including depression, sleep quality, and quality of life. Statistical significance was set at P < 0.05.

## 4. Results

This study provides a comprehensive overview of anesthesiology residents affiliated with Shahid Beheshti University of Medical Sciences. The participants, with an average age of 35.9 years, demonstrated diverse demographic characteristics, with a mean BMI of 26.01. The majority were female (60.8%) and lived in the same city where they trained (79.6%). Single individuals composed the largest marital status group (51.0%), and most did not have children (80.4%). The income distribution varied widely, with a significant portion earning less than 100 million Iranian Rials (IRRs). Despite owning private property for residency (68.6%), many respondents received financial (56.9%) and emotional support (78.4%) from their parents (Table 1). These insights contribute to understanding the challenges and needs of anesthesiology residents in the workplace.

**Table 1.** Demographic Characteristics

Variables	Values ^a^
**Age**	35.9 ± 6.7 [33 (28 - 56)]
**BMI**	26.01 ± 3.31 [26.3 (19.57 - 33.69)]
**Gender**	
Male	20 (39.2)
Female	31 (60.8)
**Identical location of residence and education**	
No	10 (20.4)
Yes	39 (79.6)
**Marriage status**	
Single	26 (51.0)
Married	22 (43.1)
Divorced	3 (5.9)
**Children number**	
0	41 (80.4)
1	2 (3.9)
2	8 (15.7)
**Monthly income from residency program (IRRs)**	
< 100	27 (52.9)
100 - 150	21 (41.2)
> 150	3 (5.9)
**Total income (IRRs)**	
< 100	24 (47.1)
100 - 150	19 (37.3)
150 - 200	2 (3.9)
200 - 500	1 (2.0)
500 - 1000	2 (3.9)
> 1000	3 (5.9)
**Residency status**	
Students accommodation	1 (2.0)
Rental	15 (29.4)
Private property	35 (68.6)
**Live with parents**	
No	33 (64.7)
Yes	18 (35.3)
**Financial support from parents**	
No	22 (43.1)
Yes	29 (56.9)
**Emotional support from family**	
High	40 (78.4)
Moderate	9 (17.6)
Less	2 (3.9)
**Night shifts per month (d)**	
4 - 6	9 (17.6)
7 - 8	13 (25.5)
9 - 10	17 (33.3)
> 10	12 (23.5)
**Working time per week (h)**	
40 - 60	5 (9.8)
60 - 80	7 (13.7)
> 80	39 (76.5)
**Smoking per day**	
No	40 (78.4)
1 - 5	2 (3.9)
6 - 10	3 (5.9)
11 - 15	3 (5.9)
16 - 20	0 (0)
> 20	3 (5.9)
**Parents education**	
Under diploma	2 (3.9)
Diploma	8 (15.7)
Advanced diploma	4 (7.8)
Bachelor	10 (19.6)
Master	12 (23.5)
PhD	4 (7.8)
General practitioner	3 (5.9)
Specialist	4 (7.8)
Subspecialist	4 (7.8)

Abbreviation: IRRs: Iranian Rials.

^a^ Values are expresses as mean ± SD [median (range)] or No. (%).

Table 2 provides a detailed examination of sleep-related traits. Regarding sleep apnea, assessed with the STOP-Bang Questionnaire, the average score was 1.1 ± 0.92, with 96.1% classified as low risk and 3.9% classified as intermediate risk. Notably, no participants were categorized as high-risk. For the ESS, the mean score was 11.82 ± 3.99, with participants classified into normal (31.4%), mild (47.1%), moderate (13.7%), and severe (7.8%) daytime excessive sleepiness categories. These findings highlight the prevalence of sleep-related issues among anesthesiology residents and the importance of targeted interventions to enhance their sleep quality and overall well-being.

**Table 2.** Sleep-Related Characteristics

Variables	Values ^a^
**STOP-Bang score**	1.1 ± 0.92 [1 (0 - 4)]
**ESS score**	11.82 ± 3.99 [12 (2 - 20)]
**Categories of STOP-Bang Questionnaire**	
Low risk	49 (96.1)
Intermediate risk	2 (3.9)
High risk	0 (0.0)
**Categories of ESS**	
Normal	16 (31.4)
Mild	24 (47.1)
Moderate	7 (13.7)
Severe	4 (7.8)

Abbreviation: ESS, Epworth Sleepiness Scale.

^a^ Values are expresses as mean ± SD [median (range)] or No. (%).

The MBI evaluated occupational burnout among participants (Table 3). Emotional exhaustion presented a mean score of 29.14 ± 8.28, with burnout levels classified as low (5.9%), moderate (52.9%), and high (41.2%). Personal accomplishment yielded a mean score of 30.55 ± 7.32, with most residents experiencing high burnout (66.7%), followed by moderate (27.5%) and low (5.9%) levels. Depersonalization recorded the highest concern, with a mean score of 39.14 ± 12.36, and all participants (100%) were classified as experiencing high burnout. These outcomes highlight the widespread presence of burnout, underscoring the urgent need for effective strategies to alleviate burnout and promote psychological well-being in this demanding medical field.

**Table 3.** Results for Three Dimensions of the Maslach Burnout Inventory

Variables	Values ^a^
**Emotional exhaustion**	29.14 ± 8.28 [28 (11 - 56)]
**Personal accomplishment**	30.55 ± 7.32 [31 (11 - 44)]
**Depersonalization**	39.14 ± 12.36 [38 (17 - 66)]
**Emotional exhaustion**	
Low burnout	3 (5.9)
Moderate burnout	27 (52.9)
High burnout	21 (41.2)
**Personal accomplishment**	
Low burnout	3 (5.9)
Moderate burnout	14 (27.5)
High burnout	34 (66.7)
**Depersonalization**	
Low burnout	0 (0.0)
Moderate burnout	0 (0.0)
High burnout	51 (100.0)

^a^ Values are expresses as mean ± SD [median (range)] or No. (%).

Table 4 presents the results of the depression test, revealing insights into participants' mental health. The mean score was 12 ± 6.24, indicating notable variation. Categorization based on scores showed a wide range of participants in all groups, with 17.65% experiencing severe depression, while the same percentage exhibited symptoms of moderate-severe depression. Additionally, 21.57% of the participants fell into the moderate depression category. These findings highlight the significant prevalence of depression among anesthesiology residents, stressing the necessity for targeted interventions and support systems to address mental health concerns within the profession.

**Table 4.** Depression Test Results of the Patient Health Questionnaire-9

Variables	Values ^a^
**Depression severity**	12 ± 6.24 [11 (1 - 23)]
**Depression categories**	
Minimum depression	6 (11.76)
Mild depression	16 (31.37)
Moderate depression	11 (21.57)
Moderate-severe depression	9 (17.65)
Severe depression	9 (17.65)

^a^ Values are expresses as mean ± SD [median (range)] or No. (%).

The results of the WHOQOL-BREF Questionnaire highlight the importance of addressing various dimensions of well-being in anesthesiology residents. These findings emphasize the need for holistic interventions aimed at enhancing both their overall health and workplace satisfaction (Table 5). 

**Table 5.** World Health Organization Quality of Life Questionnaire Results with Dimensions

Variables	Mean ± SD	Median (Range)
**Physical**	40.90 ± 14.57	42.86 (7.14 - 75)
**Psychological**	50.00 ± 17.12	50 (16.67 - 83.33)
**Social relationships**	58.33 ± 23.14	50 (0 - 100)
**Socioenvironmental**	48.47 ± 13.03	50 (25 - 75)
**Overall quality of life and general health**	60.05 ± 21.22	62.5 (25 - 100)

Exploratory post-hoc analyses were conducted to examine potential gender differences in burnout, depression, and sleep quality. Independent samples *t*-tests revealed no statistically significant differences between male and female residents in emotional exhaustion (P = 0.23), depersonalization (P = 0.52), personal accomplishment (P = 0.59), depression (P = 0.55), or sleep quality (P = 0.42). However, small effect sizes (Cohen’s d < 0.35) suggested trends toward higher emotional exhaustion and depression scores among female residents. These findings should be interpreted cautiously due to the study’s limited sample size and statistical power.

The correlations between the "Depression test" and three variables — "Emotional Exhaustion", "Personal Accomplishment" and "Depersonalization" — revealed varying levels of association. A strong positive correlation of 0.527 with emotional exhaustion was statistically significant (P < 0.001), while a moderate positive correlation of 0.484 with personal accomplishment was also significant (P < 0.001). In contrast, the correlation with depersonalization was weaker at 0.239 and did not reach statistical significance (P = 0.091).

In examining relationships with WHO.QOL, a weak and non-significant negative correlation (-0.153, P = 0.283) was found with emotional exhaustion. However, a strong negative correlation of -0.640 with personal accomplishment was statistically significant (P < 0.001). Additionally, WHO.QOL showed a moderate negative correlation of -0.434 with depersonalization, which was also significant (P = 0.001). All correlations were calculated based on a sample of 51 participants.

The correlation analysis explored the relationships between emotional exhaustion, personal accomplishment, and depersonalization with five domains: Physical, psychological, social relationships, socio environmental, and health. Emotional exhaustion demonstrated weak, non-significant correlations across all domains, such as with physical (r = -0.082, P = 0.568), psychological (r = -0.020, P = 0.890), and social relationships (r = -0.187, P = 0.188).

In contrast, personal accomplishment exhibited strong, significant negative correlations across multiple domains: Physical (r = -0.469, P = 0.001), psychological (r = -0.426, P = 0.002), social relationships (r = -0.554, P < 0.001), socio environmental (r = -0.530, P < 0.001), and health (r = -0.474, P < 0.001). These results suggest that lower personal accomplishment is associated with poorer outcomes in these areas of quality of life.

Depersonalization also showed moderate and significant negative correlations with psychological (r = -0.333, P = 0.017), social relationships (r = -0.368, P = 0.008), and socio environmental (r = -0.492, P < 0.001). The correlation with health (r = -0.242, P = 0.087) was weaker and not statistically significant. These findings suggest that higher depersonalization is linked to poorer quality of life, particularly in psychological, social, and environmental domains.

The correlation analysis between the WHOQOL-BREF and depression test with five domains — physical, psychological, social relationships, socio environmental, and health — yields the following results:

1. The WHO.QOL shows strong, statistically significant positive correlations with all domains: Physical (r = 0.781, P < 0.001), psychological (r = 0.704, P < 0.001), social relationships (r = 0.844, P < 0.001), socio environmental (r = 0.799, P < 0.001), and health (r = 0.690, P < 0.001). These results indicate that higher quality of life is associated with better outcomes across all domains.

2. Depression test shows weak and non-significant negative correlations with the physical (r = -0.135, P = 0.345) and psychological (r = -0.102, P = 0.478) domains, suggesting little to no association between depression and these areas. However, there are moderate, statistically significant negative correlations with social relationships (r = -0.419, P = 0.002), socio environmental (r = -0.410, P = 0.003), and health (r = -0.421, P = 0.002), indicating that higher depression scores are associated with poorer outcomes in these domains.

The correlation analysis between the STOP-Bang score and the ESS score with emotional exhaustion, personal accomplishment, and depersonalization provides the following results:

1. STOP-Bang score shows weak, non-significant negative correlations with both emotional exhaustion (r = -0.086, P = 0.550) and personal accomplishment (r = -0.097, P = 0.498). These results suggest little to no relationship between the STOP-Bang score and either emotional exhaustion or personal accomplishment.

2. The ESS score has a strong, statistically significant positive correlation with emotional exhaustion (r = 0.470, P < 0.001), indicating that higher sleepiness is associated with greater emotional exhaustion. The correlation with personal accomplishment (r = 0.147, P = 0.302) is weak and not statistically significant.

3. The analysis revealed a significant positive correlation between depersonalization and the ESS score (r = 0.298, P = 0.034), while no significant correlation was found with the STOP-Bang score (r = -0.103, P = 0.472).

An additional analysis of emotional exhaustion revealed several correlations between demographic factors and the variable "Emotional Exhaustion". A significant negative correlation was identified between emotional support and emotional exhaustion (r = -0.369, P = 0.008). Conversely, other variables, including primary residence, marital status, number of children, monthly and total income, living situation, cohabitation with parents, financial support, monthly shifts, weekly working hours, smoking status, and parental education, did not demonstrate significant correlations with emotional exhaustion (P > 0.05).

In summary, the statistical analyses revealed significant associations between burnout dimensions and key study variables. Emotional exhaustion showed a strong positive correlation with depression (r = 0.527, P < 0.001) and daytime sleepiness (r = 0.470, P < 0.001), while personal accomplishment was strongly negatively correlated with quality of life across multiple domains (r = -0.469 to -0.554, P < 0.001). Depersonalization also demonstrated moderate negative correlations with psychological and social quality of life (r = -0.333 to -0.492, P < 0.05). Additionally, depression was moderately negatively correlated with social relationships (r = -0.419, P = 0.002) and socio environmental quality of life (r = -0.410, P = 0.003).

These findings highlight the multifaceted nature of burnout and its broad impact on mental health, sleep quality, and overall well-being among anesthesiology residents.

## 5. Discussion

The present study aimed to explore the factors influencing overall occupational burnout among anesthesiology residents. By leveraging the results, a logical and effective approach can be developed to prevent this complication, thereby enhancing the well-being of medical practitioners and their families. Furthermore, it ensures patients’ well-being under the care and support of such graduates.

The findings of this study on anesthesiology residents provide valuable insights into the complex interplay between burnout, depression, quality of life, and sleepiness. Emotional exhaustion was strongly correlated with depression, reaffirming the link between emotional strain and depressive symptoms observed in other studies on healthcare professionals. Similarly, personal accomplishment showed a moderate positive relationship with depression, suggesting that feelings of reduced achievement may amplify depressive tendencies. Interestingly, depersonalization exhibited a weaker and non-significant association with depression, which aligns with prior research indicating that depersonalization may not always directly reflect depressive states but instead represents a distinct burnout dimension (23, 24).

Moreover, stress-induced inflammatory processes are implicated in this relationship. Psychological stress can activate inflammatory responses, increasing levels of cytokines such as IL-6 and TNF-α. These inflammatory changes have been associated with somatic and cognitive-affective symptoms of depression, highlighting a physiological pathway linking chronic stress and depressive states (25, 26).

Regarding quality of life, emotional exhaustion showed no significant relationship, which contrasts with studies that often report a notable impact of exhaustion on life quality (27, 28). However, personal accomplishment and depersonalization were both significantly associated with poorer quality of life across multiple domains, emphasizing the pervasive impact of these burnout components on well-being. These results are consistent with findings in similar research where burnout negatively affects interpersonal, psychological, and environmental quality of life dimensions (27, 29, 30).

One of the findings highlighted the concern regarding the sleep status of the residents. Although the majority of the study subjects did not primarily face sleep deprivation, it is crucial to closely monitor the sleep and rest conditions of residents under work pressure to mitigate the effects of mental exhaustion among them (31). Sleepiness is a common symptom of burnout, often characterized by fatigue, difficulty concentrating, reduced alertness, and frequent lapses into unintentional sleep. These symptoms can also result from underlying medical conditions such as sleep disorders, depression, hypothyroidism, or chronic fatigue syndrome, which may contribute to or exacerbate burnout. In this study, we used these criteria to differentiate between sleepiness caused by burnout and that stemming from other illnesses. This approach ensures a more accurate understanding of the relationship between burnout and sleepiness while minimizing confounding factors, allowing us to better target burnout-specific interventions.

In exploring sleepiness, the study revealed a strong association between higher emotional exhaustion and increased sleepiness, reinforcing existing evidence that chronic exhaustion disrupts sleep quality. Depersonalization also showed a significant relationship with sleepiness, suggesting that detachment may contribute to or result from poor sleep, as supported by prior literature (30, 32).

Emotional support was strongly associated with lower emotional exhaustion, suggesting its potential role in mitigating burnout among healthcare professionals. Individuals who received greater emotional support tended to report lower levels of emotional exhaustion, emphasizing the protective role of supportive interpersonal relationships in managing stress and emotional strain. This aligns with existing literature suggesting that social and emotional resources are critical in buffering the adverse effects of demanding professional environments (33, 34).

Interestingly, other demographic and situational variables, such as primary residence, marital status, number of children, monthly and total income, living situation, cohabitation with parents, financial support, monthly shifts, weekly working hours, smoking status, and parental education, did not show significant associations with emotional exhaustion in this study. These findings suggest that while external factors might contribute to stress, they may not directly influence emotional exhaustion in the absence of supportive mechanisms.

Consistent with prior studies, burnout is highly prevalent among residents (35). Moreover, in line with the findings of this research and previous global studies, the field of anesthesiology is identified as one of the most high-risk medical specialties due to the critical nature of patient health during surgeries and the significant psychological pressure faced by both the physician and the residents in these scenarios (5, 33). In addition, daily confrontations by other health professionals in a surgical ward dramatically add to this tension.

According to the MBI, all participants in this study were significantly affected by career burnout, confirming previous findings (5, 36). The high prevalence of burnout and depression among residents underscores the necessity for urgent interventions to enhance their well-being and avert adverse outcomes such as suicide (4, 6, 35, 37). Consistent with the findings of this study, similar research conducted by colleagues has also confirmed that the relatively low income of residents compared to that of specialists, coupled with the high levels of pressure and demanding work shifts relative to their colleagues, significantly contributes to the development of career burnout and an increased risk of depression. Sleep disorders were also identified as a key contributor to career burnout. While approximately half of the participants in the study did not experience this disorder, the pressure and stress resulting from it affected all participants in the study (35, 38).

In the field of anesthesiology residency, where stress is prevalent throughout various stages, starting from the residency exam to subsequent work shifts, support from family and teaching teams within this group can alleviate this pressure (39-41). This study reaffirmed previous findings on burnout among anesthesiology residents, particularly in anesthesiology. Based on the study by Dabbagh et al., which was conducted over four years on anesthesiology residents, it is noteworthy that many residents were trained in stress and pressure management within the scope of this study. It is essential to maintain a continuous reminder of this practice post-training to ensure sustained positive performance among residents (40, 42). In particular, these residents also experienced the COVID-19 pandemic and articulated their apprehension regarding the decrease in hands-on experience in anesthesiology, along with their uncertainty regarding their preparedness for autonomous clinical practice post-residency, a topic that has also been scrutinized within various disciplines (43).

To address burnout among anesthesiology residents, it is essential to propose context-specific solutions that align with the unique challenges faced by this group. One potential solution is to foster collaborations with national medical councils to develop and implement policies that promote healthier work environments, such as regulating work hours, mandating rest periods, and ensuring adequate emotional support for residents. Additionally, leveraging existing educational platforms to create tailored training programs could provide residents with essential skills in stress management, resilience, and time management. These programs can be integrated into current curricula, ensuring that residents receive ongoing support throughout their training. By combining policy changes with educational initiatives, it is possible to create a more sustainable and supportive environment that reduces burnout risks and enhances the overall well-being of residents.

While this study focused on anesthesiology residents, the findings align with research in other high-stress medical specialties, such as emergency medicine and surgery. For instance, studies in emergency medicine have similarly reported high rates of burnout, driven by factors like long shifts, sleep deprivation, and emotional exhaustion (44, 45). Surgical residents also face comparable challenges, including high-stakes decision-making and demanding workloads, which contribute to burnout and depression (46, 47). However, anesthesiology residents may experience unique stressors, such as the constant vigilance required during procedures and the need to manage acute, life-threatening situations. These distinctions suggest that while burnout is a pervasive issue across high-stress specialties, the specific drivers and manifestations may vary.

The limitations of this study include its small sample size, which may limit the generalizability of the findings to a broader population of anesthesiology residents or other medical specialties. Although exploratory post-hoc analyses revealed no significant gender differences in burnout, depression, or sleep quality, the small effect sizes observed suggest potential trends that warrant further investigation in larger, gender-balanced cohorts. Given the specific focus on anesthesiology residents, this research’s scope is limited to the community of Iranian anesthesiology residents, and the results may not fully reflect the experiences of residents in other regions or specialties. Additionally, the cross-sectional design of the study restricts the ability to infer causal relationships between burnout and its associated factors, as it provides only a snapshot of residents’ well-being at a single point in time. Longitudinal studies would be needed to better understand the long-term effects of burnout and its progression over the course of residency training.

Further in-depth investigations are necessary to comprehend and encompass other disciplines, providing a more comprehensive understanding of burnout across different medical fields. Another consideration is the potential variability in residents’ baseline mental health status prior to residency training, which could influence burnout outcomes. While our study did not assess pre-residency psychological states, the homogeneous nature of the cohort — all participants were anesthesiology residents exposed to comparable institutional workloads and stressors — helps mitigate confounding from external factors. Furthermore, the use of validated instruments (e.g., MBI, PHQ-9) ensured standardized measurement of burnout and depression, reducing variability in self-reported outcomes. Our statistical adjustments for demographic and situational variables, such as emotional support and sleep quality, further isolated residency-specific pressures as key contributors to burnout. However, the absence of longitudinal data limits our ability to discern whether burnout arises de novo during residency or is exacerbated by pre-existing vulnerabilities. Future studies incorporating pre-residency mental health screenings and follow-up assessments would clarify these dynamics, enabling targeted interventions for at-risk individuals.

Overall, the results highlight the multifaceted nature of burnout and its broad implications on mental health, quality of life, and sleep. These findings align with previous studies while also shedding light on specific relationships that warrant further investigation, particularly regarding the distinct roles of burnout components like depersonalization. Addressing these factors in anesthesiology residents may require targeted interventions aimed at emotional support, mental health, and sleep quality to mitigate burnout and its associated consequences.

### 5.1. Conclusions

The present study sheds light on the high prevalence of burnout among anesthesiology residents in Iran, emphasizing its detrimental impact on mental health, sleep quality, and overall quality of life. By exploring burnout through validated, culturally adapted instruments, the research provides a comprehensive understanding of its dimensions and contributing factors within the unique context of Iran, including mandatory service obligations and the growing trend of physician emigration. The findings highlight the urgent need for targeted interventions to address emotional exhaustion, promote emotional support, and mitigate stressors specific to the field of anesthesiology.

This study fills a critical gap in the existing literature by focusing on anesthesiology residents in Iran, a population facing distinctive challenges compared to their international counterparts. Future research should build upon these findings by conducting longitudinal studies to examine the progression of burnout over time and its long-term implications. Comparative analyses across medical specialties could further elucidate whether anesthesiology residents experience disproportionately higher rates of burnout and identify shared or unique stressors across disciplines. Such efforts are essential for developing robust, evidence-based interventions to improve the well-being of residents and ensure high-quality patient care.

## Data Availability

The dataset used in the study is available upon request from the corresponding author during submission or after publication.
